# Correlated evolution in parental care in females but not males in response to selection on paternity assurance behaviour

**DOI:** 10.1111/ele.12284

**Published:** 2014-04-28

**Authors:** Megan L Head, Camilla A Hinde, Allen J Moore, Nick J Royle

**Affiliations:** 1 Centre for Ecology and Conservation, College of Life and Environmental Sciences, University of ExeterCornwall Campus, Penryn, TR10 9EZ, United Kingdom; 2 Department of Animal Sciences, Wageningen UniversityWageningen, 6700AH, The Netherlands; 3 Department of Genetics, University of GeorgiaAthens, Georgia, 30602, USA

**Keywords:** Parental care, sexual conflict, co-evolution, artificial selection, *Nicrophorus vespilloides*

## Abstract

According to classical parental care theory males are expected to provide less parental care when offspring in a brood are less likely to be their own, but empirical evidence in support of this relationship is equivocal. Recent work predicts that social interactions between the sexes can modify co-evolution between traits involved in mating and parental care as a result of costs associated with these social interactions (i.e. sexual conflict). In burying beetles (*Nicrophorus vespilloides),* we use artificial selection on a paternity assurance trait, and crosses within and between selection lines, to show that selection acting on females, not males, can drive the co-evolution of paternity assurance traits and parental care. Males do not care more in response to selection on mating rate. Instead, patterns of parental care change as an indirect response to costs of mating for females.

## Introduction

Promiscuity and parenting are inextricably linked (Trivers 1972; Sheldon 2002; Alonzo 2010). Parental care is beneficial to offspring but can often be costly for parents due to energetic costs, for example, or because caring parents may forgo other reproductive opportunities (Kokko & Jennions 2008). Individuals are therefore expected to invest more care in young to which they are related (Hamilton 1964; Trivers 1972; Alonzo & Klug 2012). Mating with multiple males by females means that males typically have a lower probability of parentage than females in any given batch of offspring (Alonzo & Klug 2012). As a result, classical parental care theory predicts that, all else being equal, males should provide less parental care when offspring are less likely to be their own (Trivers 1972; Sheldon 2002; Kokko & Jennions 2008). However, empirical evidence in support of this prediction is equivocal (Alonzo & Klug 2012; Kamel & Grosberg 2012; Griffin *et al.* 2013) suggesting that all else is often not equal. For example, although counter-intuitive, the relationship between the probability of paternity and paternal effort in the current reproductive attempt may be negative rather than positive (Queller 1997; Houston & McNamara 2002; Kokko & Jennions 2008). Understanding why this might be so requires examining more than just correlations between parentage and parental effort – it requires accounting for the effects of social interactions between the sexes on the co-evolutionary relationship between traits involved with mating and those involved in parental care (Alonzo 2010).

Evolutionary conflicts of interest between parents arise because the interacting individuals are typically unrelated and there are costs of parental effort, so traits that maximise the fitness of males may not maximise the fitness of females (Lessells 2012). The resolution of such conflict has important feedback effects on the relationship between mating and parental care behaviours (Trivers 1972; Parker *et al.* 2002; Kokko & Jennions 2008; Alonzo & Klug 2012). As a result, despite considerable empirical research on different, specific components of mating and parental care interactions variation between individuals in traits such as condition or age and variation in with whom they interact can make it difficult to predict how paternity assurance traits and parental care traits co-evolve (Alonzo 2010).

Parentage is an outcome of social interactions between and within the sexes, so cannot itself evolve (Alonzo & Klug 2012). However, behaviours that provide information about expected parentage can evolve and are predicted to affect the relationship between mating and parental care (Kokko 1999; Kokko & Jennions 2008; Alonzo & Klug 2012). Behavioural interactions between the sexes during mating and parental care may be particularly important in determining the relationship between parentage and male parental care in species where both sexes care for offspring, especially when females provide the majority of care, which is most common even where both sexes provide care (Kokko & Jennions 2008). This is because male mating traits are often costly for females (e.g. seminal fluids, genital spines or high mating rates; reviewed in Chapman *et al.* 2003; Arnqvist & Rowe 2005; Chapman 2006). If these costs of mating are great enough to affect the ability of females to provide care and reduce offspring performance, behaviours that increase male parentage (paternity assurance), such as repeated mating, will determine how selection acts on patterns of parental care. Such dynamic co-evolutionary relationships between traits, mediated by social interactions between males and females, can only be quantified effectively using experimental evolution, to test whether selection on traits involved in mating drive predictable changes in parental care of males and females (Alonzo 2010).

Here we use artificial selection on a paternity assurance trait (repeated mating rate) in burying beetles *Nicrophorus vespilloides*, to investigate how social interactions between the sexes during mating co-evolve with parental care. Parental care in *N. vespilloides* involves direct feeding of offspring and indirect care involving the maintenance and defence of breeding resources (Walling *et al.* 2008). This species facultatively expresses all forms of care (uniparental female care, uniparental male care, and biparental care), the amount of which is highly variable and positively affects offspring fitness (Eggert *et al.* 1998; Smiseth *et al.* 2003; Lock *et al.* 2004; Walling *et al.* 2008). Multiple paternity of broods is the norm in burying beetles (Eggert 1992; House *et al.* 2009) and females may provide more direct care than males (Smiseth *et al.* 2005; Walling *et al.* 2008). There is fierce male–male and female–female competition for the carcasses upon which burying beetles breed. During carcass preparation the dominant male engages in mate guarding, and repeatedly mates with the female (Eggert 1992). High repeated mating rates are associated with increased levels of paternity for males (Müller & Eggert 1989; House *et al.* 2007) but mating more than twice provides no direct benefit to females (House *et al.* 2008, 2009).

Using artificial selection allows us to control for within-sex social interactions during mating and parental care, so that we can specifically quantify how between-sex social interactions (with potential for conflict) influence the co-evolution of mating and parental care behaviours. Following artificial selection on repeated mating rate for six generations we conducted mating crosses within and between divergent selection lines to determine how selection regime (H = lines selected for high repeated mating rate, L = lines selected for low repeated mating rate) affected mating and parental behaviour and offspring performance of individuals in relation to the selection regime of their partner. We predict that if there is direct, positive co-evolution between mating behaviour and paternal effort (i.e. males evolve to invest more in offspring when they have a higher perceived probability of parentage) then males from lines selected for high repeated mating rate will provide more care and have higher offspring performance in the line crosses than males from low lines (i.e. H > L). Alternatively, if there is no direct co-evolution between mating and parental care behaviours then we predict male selection regime will have no significant effect on paternal care behaviour (i.e. H = L). In addition, if mating is sexually antagonistic this will lead to different fitness optima for males and females so it is predicted that females from high lines will have lower trait values for parental care and offspring performance than females from low lines (i.e. L > H), resulting from costs of mating.

We find that patterns of parental care evolve in response to selection on repeated mating rate. However, this is via indirect effects on maternal care, not direct effects on paternal care: costs of mating in females, not paternity assurance in males, explain the co-evolutionary relationship between mating and parental care behaviours in *N. vespilloides*. Consideration of costs of maternal care, resulting from sexual conflict over mating rate, not just costs of care to males (*sensu* Griffin *et al.* 2013), may help explain variation in patterns of parental care within and between species.

## Materials and Methods

### Experimental design

Beetles used in this experiment were obtained from generation F7 of lines selected for high and low re-mating rates (see supplementary information for details of selection regime and origin and maintenance of beetles). Control lines were not included in this experiment. To examine co-evolution of male and female mating and parental care behaviour we set up 15–20 trials of all potential combinations of line crosses including within lines, across lines within treatments and across treatments (i.e. a total of 275 trials distributed evenly across 16 cross-types (Table S1)).

### Mating behaviour

For each cross we conducted a mating trial where we recorded both male and female mating behaviour for 1 h. Mating trials were conducted in a Petri dish (8.5 cm diameter) lined with filter paper. For each trial we first placed a virgin female in the Petri dish. The female was allowed to acclimate for 2 min before a virgin male was added to the Petri dish. We began recording male and female mating behaviour using Observer software (Noldus Information Technology, Wageningen, Netherlands) as soon as the male was introduced. During mating trials, we recorded the number of matings defined as the number of times a male inserted his aedeagus into the female, as well as the proportion of matings that were preceded by female resistance. Female resistance consists of three different behaviours: wrestling, which prevents the male from mounting; kicking, which can sometimes dislodge the male from the females back; and abdomen curling, which prevents the male inserting his aedeagus. These behaviours are common female resistance behaviours in insects (Perry *et al.* 2009).

To visualise the effects of selection regime on overall mating behaviour we used principal components analysis to create a composite variable that reflected the components of mating we measured: number of matings and the proportion of matings resisted. Prior to conducting principal components analysis the number of matings was power transformed to meet the assumption of normality of principal components analysis. The appropriate power transformation was determined using the Power transform function in the ‘car’ package of R (Fox & Weisberg 2011). This Principal components analysis resulted in a single significant eigenvector (PC1) that accounted for 62.7% of the variation in these mating traits. Both the number of matings and the proportion of matings resisted loaded strongly and positively on PC1 (which we call mating behaviour from here on) (loadings for both traits = 0.792).

### Parental care behaviour

For each cross we recorded both male and female duration of parental care of broods that were controlled for the number and origin of offspring. To do this, as soon as a pair completed their mating trial, we transferred them to a breeding container (17 × 11 × 6 cm) which contained 2 cm of moist soil and a mouse carcass (supplier: Livefoods Direct, Sheffield, UK) weighed to 0.01 g (mean carcass weight ± S.D. = 22.32 ± 1.26). Pairs were allowed to interact freely in the breeding container and although the frequency of mating may have decreased over time it is expected to decrease similarly across all pairs and so differences related to selection regime are likely to have been maintained. Fifty-eight hours after pairs were set up in breeding containers they and their processed carcass were removed and placed in a new breeding container. This allowed us to isolate the eggs, which are laid in the soil surrounding the carcass, before they hatched (Smiseth *et al.* 2006). At this stage, each pair's eggs were placed in individual containers with moist soil and a small amount of ground beef to prevent starvation upon hatching. Eggs were checked every 8 h to establish the onset of hatching. Hatching larvae from each pair were pooled together with all other larvae hatching at the same time. These larvae were then distributed to the carcasses of breeding pairs. Each pair received 20 larvae. Care was taken to ensure that larvae were distributed to each carcass no sooner than 59 h after a female's first eggs were laid, as parents with larvae arriving earlier than expected have previously been shown to have high rates of offspring rejection (Müller & Eggert 1990; Eggert & Muller 2000). By controlling the number of larvae added to each carcass we ensured that differences in offspring performance were due to differences in parental care and not differences in fecundity or fertility. In addition, by providing each carcass with the same number of larvae we control for effects of brood size which are known to influence parental care behaviour (Smiseth & Moore 2004).

Once larvae were added to the carcass, pairs were checked every 8 h to determine the duration of male and female care. During each check presence/absence of the male and female was recorded. The duration of care was then estimated as the time, since larval arrival, until each beetle was recorded as absent from the carcass for two consecutive observations (Benowitz *et al.* 2013). When beetles are present on the carcass they perform both direct (feeding of larvae) and indirect (maintenance of the carcass) care. When beetles are absent from the carcass they are usually found buried in the soil away from the carcass. Presence on the carcass is a strong indicator of how much care is provided to offspring (Walling *et al.* 2008). We continued to record presence or absence of adult beetles until larvae dispersed from the carcass. Therefore, the number of observations ranged between 11 and 19.

### Offspring performance

We measured offspring performance to evaluate the fitness effects of parental care. Once adult beetles had ceased parental care and abandoned the carcass we continued to monitor each breeding box every 8 h for larval dispersal. Larvae were classed as dispersing when at least two larvae had left the carcass and were wandering around on the surface of the soil or were buried in the soil away from the carcass. At dispersal we counted the number of larvae dispersing and weighed each whole brood to 0.1 mg using an Ohaus Explorer digital balance. Larval development time on the brood was measured as the time between the addition of larvae to the carcass and larval dispersal. Mean larval weight was calculated as the brood mass divided by the number of larvae dispersing. The proportion of larvae surviving was calculated as the number of larvae dispersing divided by 20 (i.e. the number of larvae added to the carcass; Head *et al.* 2012). Only one pair did not have any larvae disperse from the carcass.

We used principal components analysis to create a composite measure of offspring performance influenced by parental care (Eggert *et al.* 1998; Eggert & Muller 2000). All three offspring performance measures loaded positively on PC1 (Loadings: mean larval weight = 0.754, proportion of larvae surviving = 0.729, larval development time = 0.512), which explained 45.5% of the variation in postnatal offspring performance data. Results for each offspring performance measure analysed separately can be found in the online information supplement (Fig. S2).

### Data analysis

To determine how male and female selection regime influences mating behaviour (number of matings per hour, proportion of matings resisted and mating behaviour (PC1)), parental care (male and female duration of care) and offspring performance we performed GLMMs in R version 3.0.2 (R development team 2013). In these models, we included male selection regime (fixed) and female selection regime (fixed) as well as the interaction between these two effects. We used model selection by backward stepwise deletion, starting with the interaction term, until only significant terms (*P* < 0.05) were left, to obtain final models that provided the best fit to our data. Non-significant effects are reported as their significance at the time of removal and significant effects are reported from the final models. For response variables that were normally distributed we used the package lmerTest to obtain significance tests of *t*-values. We also included cross-type (i.e. each specific combination of line crosses) as a random effect in our model to account for pseudo-replication of our trials within each cross-type. The sample size within each cross-type is given in the online supplement (Table S1). For each response variable appropriate distributions were specified.

## Results

### Effectiveness of selection regime

**Figure 1:**
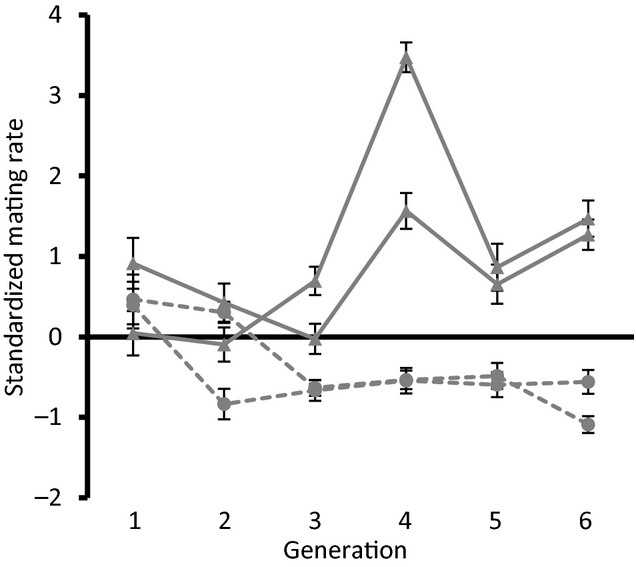
Standardised response to selection on repeated mating rate over six generations prior to the experiment. To control for environmental variation across generations we plot deviations in the mean (±SEM) repeated mating rate of selected lines from the mean repeated mating rate of the corresponding control line. Triangles with solid lines were selected for high repeated mating rate. Squares with dashed lines were selected for low repeated mating rate. Absolute data for all lines can be found in the online supplement (Fig. S1)

Bidirectional selection applied to repeated mating rate resulted in divergence between lines within six generations (Fig. 1). The responses of lines selected for both high and low repeated mating rates were significantly different from control lines. Repeated mating rate of high lines increased relative to control lines (Poisson GLMM: 3472 observations of six lines, *z* = 4.20, *P* < 0.0001), while repeated mating rate of low lines decreased relative to control lines (3472 observations of six lines, *z* = −4.33, *P* < 0.0001). This result demonstrates that our selection regime was effective at driving genetic divergence between lines.

### Mating behaviour

**Figure 2:**
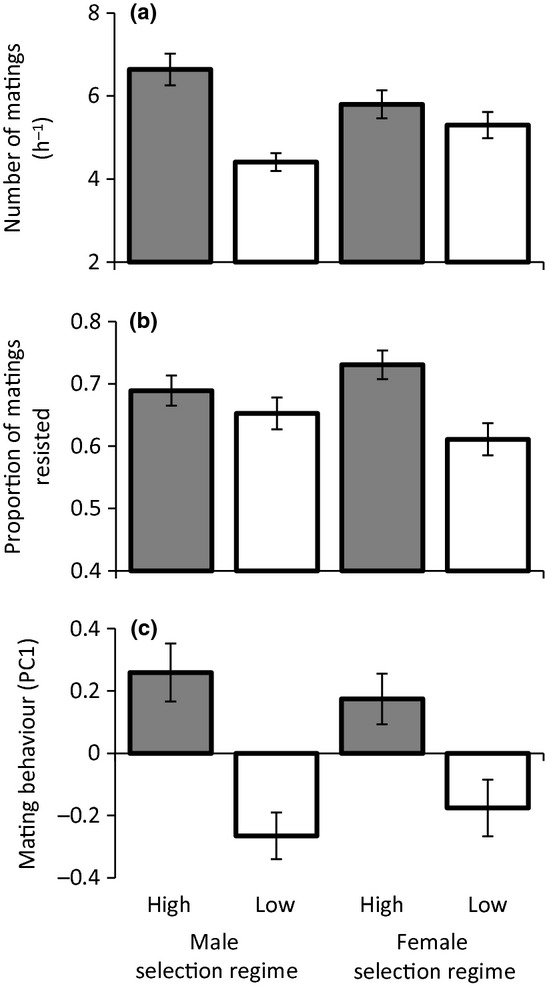
The effects of male and female selection regime on mating behaviour in *Nicrophorus vespilloides* burying beetles. Mean (±SEM). (a) The number of matings per hour. *n* = 275 observations of 16 cross-types (b) The proportion of matings resisted. *n* = 275 observations of 16 cross-types (c) The first principal component of overall mating behaviour. Grey bars correspond to crosses involving males or females from lines selected for high repeated mating rates. White bars correspond to crosses involving males or females selected for low repeated mating rates *n* = 261 observations of 16 cross-types.

The number of matings per hour was greater for crosses which included males from lines selected for high repeated mating rates (Poisson GLMM: *z* = −5.19, *n* = 275 observations of 16 cross-types, *P* < 0.001), but was not influenced by either female selection regime (*χ*^2^ = 1.538, *P* = 0.215), or the interaction between male and female selection regime (*χ*^2^ = 0.314, *P* = 0.575) (Fig. 2a). In contrast, the proportion of matings resisted was greater for crosses which included females from lines selected for high repeated mating rates (Binomial GLMM: *z* = −2.690, *n* = 275 observations of 16 cross-types, *P* = 0.007), and to a lesser extent crosses that included males from lines selected for high repeated mating rates (*z* = −2.185, *P* = 0.029) (Fig. 2b). There was also a marginally non-significant effect of the interaction between male and female selection regime on the proportion of matings resisted (*χ*^2^ = 3.169, *P* = 0.075). This may potentially be driven by the particularly low proportion of matings resisted when females from lines selected for low repeated mating rates were paired with males from the same line. Our analysis investigating the overall effects of selection regime on the PC1 of mating behaviour indicates that both male (Gaussian GLMM: *t* = −4.246, *n* = 261 observations of 16 cross-types, *P* = 0.001) and female (*t* = −2.881, *n* = 261 observations of 16 cross-types, *P* = 0.013) selection regime lead to divergence in overall mating behaviour (Fig. 2c). There was no interaction between male selection regime and female selection regime on the PC1 of mating behaviour (*χ*^2^ = 0.509, *P* = 0.476). These results indicate that divergence in mating behaviour was the result of co-evolution of male persistence and female resistance traits.

### Parental care behaviour

**Figure 3:**
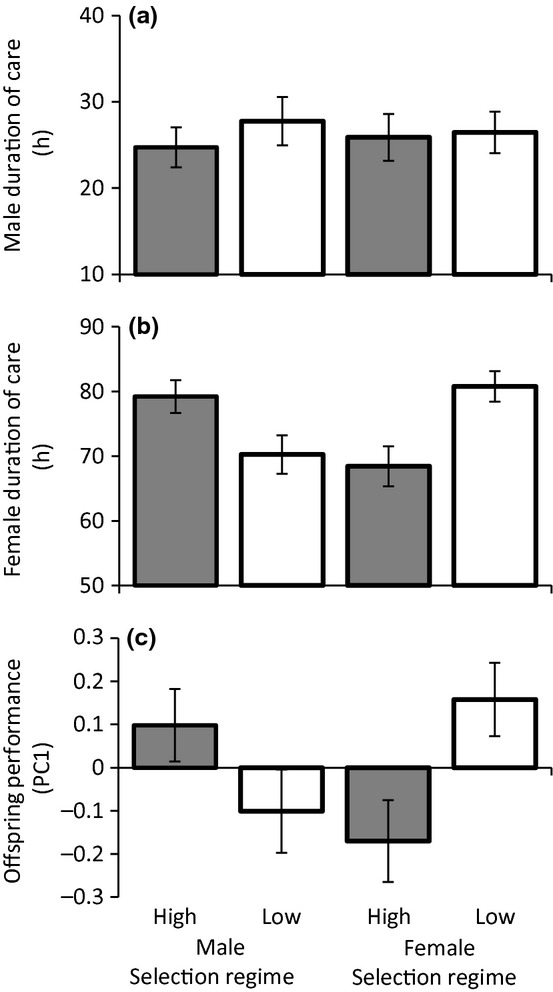
The effects of male and female selection regime on parental care behaviour and offspring performance in *Nicrophorus vespilloides* burying beetles. Mean (± SEM). (a) Male duration of care. *n* = 241 observations of 16 cross-types (b) Female duration of care. *n* = 241 observations of 16 cross-types (c) The first principal component of postnatal offspring performance (proportion of larvae surviving, mean larvae weight, larval development time). Grey bars correspond to crosses involving males or females from lines selected for high repeated mating rates. White bars correspond to crosses involving males or females selected for low repeated mating rates. *n* = 243 observations of 16 cross-types.

Despite divergence between selection regimes in male repeated mating rate we found no evidence that selection on repeated mating rate influenced male parental care behaviour. Male duration of care was unrelated to either male (Negative binomial GLMM: *χ*^2^ = 0.306, *n* = 241 observations of 16 cross-types, *P* = 0.580) or female (*χ*^2^ = 0.036, *P* = 0.849) selection regime (Fig. 3a), or the interaction between them (*χ*^2^ = 1.249, *P* = 0.264). In contrast, female selection regime explained a significant amount of the variation in female duration of care. Females from lines selected for low repeated mating rates cared for larvae longer than females from lines selected for high repeated mating rates (Negative binomial GLMM: *t* = 3.13, *n* = 241 observations of 16 cross-types, *P* = 0.002). Female duration of care was not influenced by the selection regime of males (*χ*^2^ = 2.275, *P* = 0.132) (Fig. 3b), or the interaction between male and female selection regime (*χ*^2^ = 0.495, *P* = 0.482).

### Offspring performance

**Figure 4:**
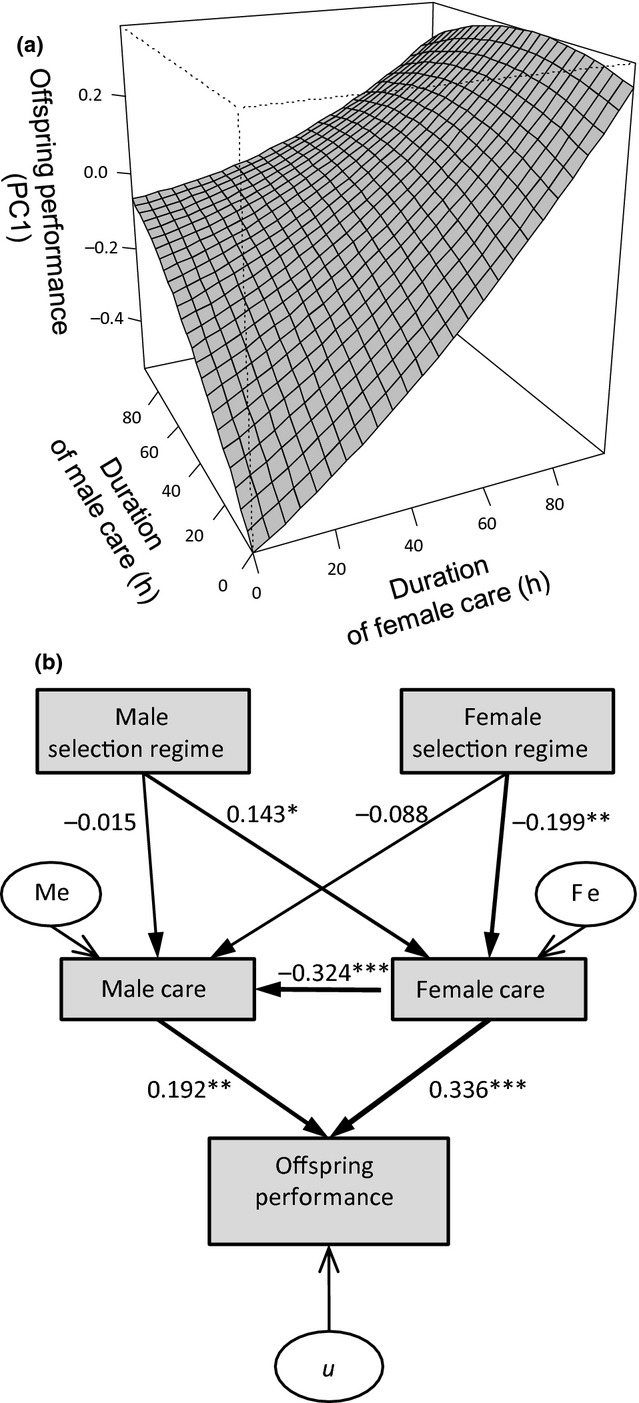
(a) Response surface showing the relationship between the duration of male and female care and postnatal offspring performance in *Nicrophorus vespilloides* burying beetles. *N* = 233 broods. We used sequential multiple regression to test for both linear and nonlinear effects and visualised this surface following the methods detailed in Lenth (Lenth 2009). (b) Results from path analysis showing direct and indirect relationships between selection regime and duration of care of males and females on offspring performance. The model used was determined a priori using information from previously published research (Smiseth *et al.* 2005). We report standardised path coefficients, because male duration of care and female duration of care are both dependent and predictor variables (Heise 1975). Direct effects of selection regime on offspring performance were not included, because we controlled for offspring number and randomised line of origin prior to larvae being added to the carcass.

Analysis of traits reflecting post-natal offspring performance qualitatively matched results obtained for female duration of care: Females from lines selected for low repeated mating rates had offspring with higher performance than females selected for high repeated mating rate (Gaussian GLMM: *t* = 2.587, *n* = 243 observations of 16 cross-types, *P* = 0.010) (Fig. 3c). Offspring performance was not related to male selection regime (*χ*^2^ = 2.338, *P* = 0.126), and the interaction between male and female selection regime was also non-significant (*χ*^2^ = 0.163, *P* = 0.687). Our multiple regression analysis indicates that this result is likely to be because duration of female care is the primary determinant of post-natal offspring performance (β ± CI = 0.307 ± 0.125, *P* < 0.001). The duration of male care also influenced post-natal offspring performance (β ± CI = 0.202 ± 0.123, *P* = 0.004), but less strongly than female duration of care (Fig 4).

## Discussion

Our results show that parental care evolves in response to selection on paternity assurance behaviour in *N. vespilloides*: specifically that female parental care decreases in lines selected for higher repeated mating rate. In contrast to expectations based on classical parental care theory (reviewed in Sheldon 2002; Alonzo & Klug 2012), however, male parental care did not evolve in response to high repeated mating rates which ensure increased paternity assurance. Instead, selection for high repeated mating rate led to reduced maternal care, most likely a result of an indirect sexually antagonistic co-evolutionary response by females to costs associated with repeated mating. In common with most species in which both males and females provide parental care (Kokko & Jennions 2012), we also find that offspring performance in burying beetles is determined primarily by variation in female parental care behaviour (see also Walling *et al.* 2008). As a result of the greater contribution of female care than male care to offspring performance, selection acting on mating behaviour in females rather than selection acting on paternity assurance in males drives co-evolution of mating and parental care behaviours: Sexual conflict is more important than parentage in explaining observed patterns of parental care in *N. vespilloides*.

Artificial selection on repeated mating rate (an interacting phenotype; see House *et al.* 2008) resulted in rapid evolutionary divergence between lines: Selection for high repeated mating rate resulted in the evolution of males with higher repeated mating rates as well as females that resisted a greater proportion of male mating attempts, with opposite results in the lines selected for low repeated mating rate. Interacting phenotypes are traits expressed in social interactions and are predicted to evolve more rapidly than standard traits because of co-evolutionary feedback where the evolving trait also exerts selection (Moore *et al.* 1997; Moore & Pizzari 2005; McGlothlin *et al.* 2010). This rapid evolution suggests that repeated mating rate in *N. vespilloides* has evolved through sexually antagonistic co-evolution. While high mating rates in burying beetles have previously been shown to be beneficial to males, increasing their likelihood of parentage (Bartlett 1988; Müller & Eggert 1989), females do not benefit from high mating rates (House *et al.* 2008) and are likely to incur costs, either directly (e.g. as a result of damage; (Arnqvist & Rowe 2005; Eady *et al.* 2007) or indirectly (e.g. through energetic costs of increased resistance (Jormalainen *et al.* 2001). Our result that females from lines selected for high repeated mating provided less parental care than low line females indicates that costs associated with high repeated mating rates compromise the ability of females to provide parental care. Furthermore, as a result of these likely costs of mating for females, patterns of parental care co-evolved indirectly with paternity assurance behaviours via effects on parental care in females.

Although male parental care has a positive effect on offspring performance during both biparental care and when males provide care for offspring alone (Walling *et al.* 2008; Head *et al.* 2012), females provide more care than males and duration of female care is the primary determinant of offspring performance, not the duration of male care (Fig. 4). As a result any factors that impact upon the ability of females to provide care are expected to have more profound consequences on the evolution of patterns of parental care than factors that impact male parental effort. Indirect costs to females, such as costs imposed by males associated with paternity assurance behaviours, are more likely to drive the evolution and maintenance of sex roles in parental care than direct benefits of such behaviours for males. Accordingly, we found that selection on repeated mating rate led to a correlated change in parental care in females, not males: the selection regime of females affects duration of care of mothers (females from lines selected for high mating rates spent less time caring than low line mothers), but male care was not significantly related to the selection regime of either males or females (Fig. 3). Although male parental effort did not respond to selection on mating behaviour, males (and females) clearly vary in their parental effort and this variation affects offspring performance (Figs.4). So what explains variation in male parental effort? In a previous study (Benowitz *et al.* 2013), we showed that male burying beetles respond behaviourally to changes in the probability of parentage during parental care in relation to their own age or condition. However, because females compensated by modifying their own parental effort in response to male status, the net result was no change in offspring performance. Social interactions between males and females during parental care can, therefore, limit the scope for direct co-evolution between paternity assurance behaviours and levels of care in males.

Our experiment demonstrates that selection on repeated mating rate leads to co-evolutionary changes in parental care, via effects on females likely resulting from costs associated with high mating rates. This result highlights the importance of social interactions during mating and parental care in determining how these traits co-evolve (Parker *et al.* 2002; Alonzo 2010). Consequently, this study provides evidence that conflicts of interest between males and females and the intensity of sexual selection may be more important than parentage in shaping sex roles in parental care. Our results also provide some support for recent theory that suggests that the key to understanding patterns of parental care is the cost of investing in mating traits relative to other traits that affect fitness, such as parental care, and not the probability of parentage *per se* (Kokko *et al.* 2012). This trade-off is shaped by how beneficial a new reproductive opportunity would be for an individual and how difficult it is to gain. The costs and benefits of seeking further reproductive opportunities for males, for example, will be affected not just by social interactions between males and females, but also by the availability of such opportunities, how much competition between males there is for them and how much choice females exercise (i.e. the intensity of sexual selection; Kokko *et al.* 2012).

By experimentally controlling for the opportunity for sexual selection our results demonstrate the importance of sexual conflict in shaping parental care evolution. However, the very aspect of our experimental design that allowed us to separate effects arising as a consequence of male–female interactions from those arising from interactions within the sexes also means that important effects arising from male–male or female–female behaviours were not quantified. For example, repeated mating behaviour may be modified by the presence of other (non-focal) individuals, so our selection regime, which involved just single male and female pairs, may have resulted in the uncoupling of repeated mating from paternity assurance behaviour as a result of a lack of social cues indicative of the probability of sperm competition (Benowitz *et al.* 2013). However, this seems unlikely because repeated mating is a mechanism of paternity assurance in burying beetles (Müller & Eggert 1989; Müller *et al*. 2007), so selection on repeated mating rate should lead to correlated evolution of any genetically correlated traits regardless of the presence of a competitor. Moreover, given that repeated mating often occurs in the wild when other (non-focal) individuals are not present (Eggert 1992), and there were only a relatively short number of generations over which selection occurred, it is difficult to conceive that the traits could be so rapidly uncoupled. It may also be that a lack of cues indicating further potential breeding opportunities elsewhere may constrain the expression of behaviour in males and females. Nevertheless, our experiment clearly demonstrates that mating behaviour and parental care co-evolve: costs of mating for females affect their ability to provide parental care, and because females provide the majority of care this leads to a reduction in offspring performance. The evidence indicates that sexual conflict over mating is more important than paternity assurance *per se* in driving the evolution of parental care in *N. vespilloides* burying beetles.

Future research on the evolution of sex roles in parental care may be better served by concentrating on quantifying the trade-offs between investment in mating vs. investment in parental care traits (Kokko *et al.* 2012), rather than simply establishing whether the probability of parentage predicts variation in parental care in males. In quantifying these trade-offs it will be important to take account of social interactions between and within the sexes. Recent theoretical work indicates that mating-parental care relationships are not unidirectional, and by providing parental care males might reduce their uncertainty of paternity, not the other way around (Kvarnemo 2006; Kahn *et al.* 2013). Our experiment selected on mating behaviour and looked for correlated responses in parental care, but showed that the two co-evolve, meaning there is feedback between the two. In the context of competition from other males for breeding resources and access to females, providing parental care may be an effective means of protecting parentage for male burying beetles. In the absence of direct competition from rival males during breeding attempts, a situation that occurs frequently in the wild in *N. vespilloides* (Hopwood, Moore and Royle unpublished data), males may modify parental care behaviour accordingly (e.g. reducing time spent on such potentially costly behaviours). These forms of social context-dependent relationships between mating and parental care behaviours may help explain the unusually flexible patterns of parental care shown by burying beetles (Scott 1998). More generally, the importance of social interactions between males and females in determining correlations between mating and parental care behaviours that we found under biparental care implies that co-evolutionary relationships between mating and parental care behaviours are likely to be very different under uniparental care.
