# Supplementary material for: Correlated evolution in parental care in females but not males in response to selection on paternity assurance behaviour
Source: Ecol Lett. 2014 Apr 28;17(7):803–10. doi: 10.1111/ele.12284 (PMC4285953; doi:10.1111/ele.12284)
Supplement: Supplementary file 2 — Supplementary [file ele0017-0803-SD2.pdf]

Head et al. SUPPLEMENTARY INFORMATION

Supplementary methods and results

Origin and maintenance of burying beetles

Approximately 90 male and 90 female *Nicrophorus vespilliodes* were collected from Devichoys Wood, Cornwall, UK (N 50°11'47''E-5°7'23'') in July, 2010. Beetles were trapped using Japanese beetle traps baited with salmon. Female burying beetles mate multiply in the field and can store sperm for long periods of time (Müller & Eggert 1989). Therefore, the effective population size, and hence genetic variation in our starting population was greater than suggested by the number of beetles collected. This stock was maintained by breeding 50-60 pairs per generation. To ensure that this stock remained outbred, pairs were created from randomly matched males and females except that siblings and first cousins were never paired. In addition, each beetle was only used in one breeding attempt per generation. To breed, a pair of virgin male and female beetles were placed in a breeding chamber (i.e. a transparent plastic container: 17 x 12 x 6cm) filled with 2 cm of moist soil and a 15-25g mouse carcass (supplier: Livefoods Direct, Sheffield), sprayed with water to aid in carcass preparation and maintenance. Larvae dispersing from these carcasses were removed from the breeding chamber and placed in individual rearing containers (clear plastic container: 7 x 7 x 4cm) filled with 2cm of moist soil. After eclosion beetles were sexed and fed 2 decapitated mealworms (*Tenebrio moliter*) twice a week until reproductive maturity (~14 days, Hopwood *et al.* 2013). All rearing was conducted in a constant temperature room at 21±1°C with a 16L:8D light regime.

Artificial Selection Regime

We derived our starting population from the third generation of laboratory reared burying beetles. This reduced the likelihood that any environmental and/or maternal effects would influence the response to selection (Lynch & Walsh 1998). For our starting population (F0), we randomly paired 107 males and females and recorded their mating rate. To measure mating rate a male and female beetle were placed in a petri dish (8.5cm diameter) lined with filter paper, and the number of matings the pair engaged in, in the following hour was recorded. Mating begins when a male inserts his aedeagus (intromittent organ) into a female vagina and ceases when it is removed (House *et al.* 2008), which is easily observed and recorded. These pairs were then set up to breed (methods outlined above). Families of larvae from these pairs were then allocated to three treatments (High, Low, Control) based on their parents mating rate. Larvae produced from pairs from the top and bottom 30% of mating rates were allocated high and low treatments respectively, while larvae contributing to the control treatment were taken from a random sample of 30% of the families. In our F1 generation (the first generation of selection) we split each of the 3 treatments into 2 replicates to create a total of 6 lines. In this generation we recorded the mating rate of 82 pairs of beetles per line and set these beetles up to breed. For each line we kept the top, bottom and random 35 families for the high, low and control treatments respectively, to contribute to the F2 generation. For F2 and all subsequent generations we paired and recorded the mating rate of 100 males and females within each line. From these we kept the top (High), bottom (Low) and a random (Control) 20-25% of families to breed and contribute to subsequent generations. This selection regime was continued for 4 further generations. For the first two generations (F0,

F1) mating was carried out over 40 days. However, in all subsequent generations mating trials for each line were carried out on a single day when beetles were between 14 and 21 days of age (except in generation 5 where beetles were mated between 21and 28 days of age). This ensured that beetles were sexually mature while minimizing age effects on mating rate. For the first two generations (F0, F1) we kept all larvae from each family that dispersed from the carcass to contribute to the next generation. From F2 onwards we kept a maximum number of larvae (that dispersed from the carcass) per family (ranging between 15 and 25). This gave us enough larvae to ensure enough adult beetles to contribute to the next generations round of mating trials and reduced the likelihood of inadvertent selection on traits correlated with reproductive success such as female size. Males and females to be used in mating trials in each generation were selected from the available pool of adult beetles and paired randomly (with the exception that brothers and sisters were never paired).

Table S1. Distribution of replicates across the different cross types

		**Male line**			
		**High1**	**High2**	**Low1**	**Low2**
**Female line**	**High1**	18	17	16	20
	**High2**	17	17	15	17
	**Low1**	17	19	16	17
	**Low2**	17	18	17	17

References

Hopwood, P.E., Moore, A.J. & Royle, N.J. (2013) Nutrition during sexual maturation affects competitive ability but not reproductive productivity in burying beetles, *Funct. Ecol.*, 27, 1350-1357.

House, C.M., Evans, G.M.V., Smiseth, P.T., Stamper, C.E., Walling, C.A. & Moore, A.J. (2008) The evolution of repeated mating in the burying beetle, *Nicrophorus vespilloides*, *Evolution*, 62, 2004-2014.

Lynch, M. & Walsh, B. (1998) *Genetics and analysis of quantitative traits*, Sinauer Associates, Sunderland, Massachusetts, USA.

Müller, J.K. & Eggert, A.-K. (1989) Paternity assurance by helpful males - adaptations to sperm competition in burying beetles, *Behav. Ecol. Sociobiol.*, 24, 245-249.

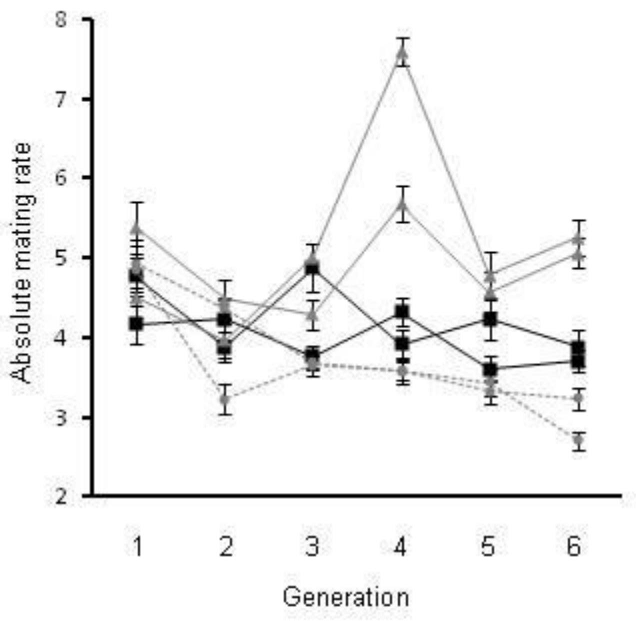

Figure S1. Response to selection on repeated mating rate. Absolute mating rate values (mean ± SEM) are presented for each of the 6 selection lines over 6 generations of selection. Triangles with solid grey lines represent lines selected for high repeated mating rate, circles with dashed grey lines represent lines selected for low repeated mating rate and squares with black lines represent control lines. Statistical analyses showing significant differences between selection regimes in response to selection are presented in the main manuscript.

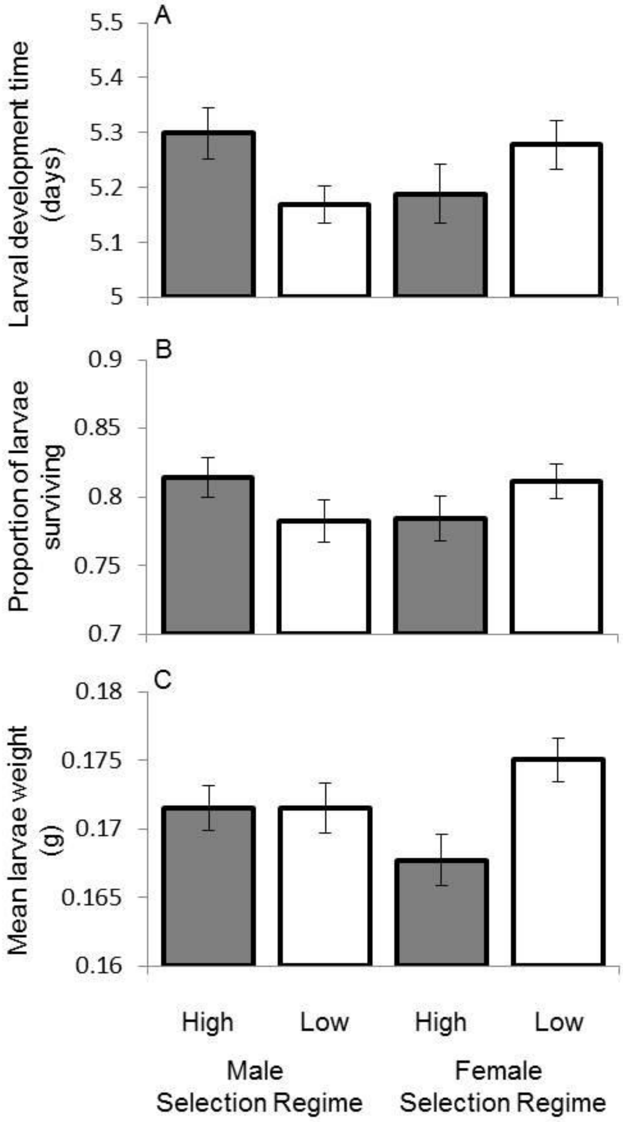

Figure S2. Effects of male and female selection regime on individual offspring performance components. A) Larval development time: no effect of male selection regime (negative binomial GLMM: 243 observations across 16 cross-types, $\chi^2 = 0.1966$, $p = 0.6575$), female selection regime ($\chi^2 = 0.0872$, $p = 0.7678$) or the interaction between male and female section regime ($\chi^2 = 0.0000$, $p = 0.9889$); B) Proportion of larvae surviving until dispersal: no effect of male selection regime (binomial GLMM: 243 observations across 16 cross-types, $\chi^2 = 2.0937$, $p = 0.1479$), female selection regime ($\chi^2 = 0.6161$, $p = 0.4325$), or the interaction between male and female section regime ($\chi^2 = 0.6988$, $p = 0.4032$); C) Mean larval weight: no effect of male selection regime (Gaussian GLMM: : 243 observations across 16 cross-types, $\chi^2 = 0.002$, $p = 0.9641$), a significant effect of female selection regime ($\chi^2 = 8.9467$, $p = 0.0028$), and no interaction between male and female section regime ($\chi^2 = 0.2687$, $p = 0.6042$) Grey bars correspond to crosses involving males or females from lines selected for high repeated mating rates. White bars correspond to crosses involving males or females from lines selected for low repeated mating rates.

Alternative analysis of data

In addition to the GLMM approach taken in the main text we also conducted a more conservative analysis which pooled data for pairs belonging to each specific type of line cross. This analysis gave qualitatively similar results to the GLMMs. Details and results of this analysis are provided below (Table S2).

Data analysis

To determine how male and female selection regime influences mating behaviour (mating rate, proportion ofmatings resisted), parental care (male and female duration of care) and postnatal reproductive success we pooled data for pairs belonging to each specific type of line cross (Table S1) giving a total of 4 replicates of each (i.e. HH, HL, LH, LL). By pooling data within cross-types we avoid pseudo replication associated with using multiple trials within each cross-type. We then tested the effects of male selection regime, female selection regime and their interaction using analysis of variance (ANOVA) (SPSS version 20).

Results

Results using this conservative analysis support the results in our main manuscript which use a GLMM approach.

Table S2. Effects of male and female selection regime on mating behaviour, parental care and offspring performance – a conservative analysis using mean data pooled to cross-type.

Response variable	Source of effect	$F_{(1,12)}$	P
Mating rate	Male selection regime	24.173	0.000
	Female selection regime	1.504	0.244
	Interaction	0.560	0.469
Proportion of matings resisted	Male selection regime	1.246	0.286
	Female selection regime	10.979	0.006
	Interaction	3.417	0.089
Mating behaviour (PC1)	Male selection regime	16.885	0.001
	Female selection regime	8.029	0.015
	Interaction	0.519	0.485
Male duration of care	Male selection regime	0.647	0.437
	Female selection regime	0.010	0.922
	Interaction	1.092	0.317
Female duration of care	Male selection regime	4.475	0.056
	Female selection regime	8.828	0.012
	Interaction	0.899	0.362
Offspring performance (PC1)	Male selection regime	1.955	0.187
	Female selection regime	5.623	0.035
	Interaction	0.423	0.528
